# A Magnetic Beads-Based Sandwich Chemiluminescence Enzyme Immunoassay for the Rapid and Automatic Detection of Lactoferrin in Milk

**DOI:** 10.3390/foods13060953

**Published:** 2024-03-21

**Authors:** Wenjie Shen, Zhihong Xuan, Hongmei Liu, Kai Huang, Xiao Guan, Baoyuan Guo

**Affiliations:** 1 School of Health Science and Engineering, University of Shanghai for Science and Technology, Shanghai 200000, China; shenwenjie0713@163.com (W.S.); hkjn1990@163.com (K.H.);; 2 Institute of Grain and Oil Quality Safety, Academy of National Food and Strategic Reserves Administration, Beijing 100000, China

**Keywords:** lactoferrin, milk powder, magnetic nanoparticle, biotin–streptavidin system, chemiluminescence immunoassay

## Abstract

Lactoferrin (LF), an iron-binding glycoprotein with immunological properties and a high nutritional value, has emerged as a prominent research focus in the field of food nutrition. Lactoferrin is widely distributed in raw milk and milk that has undergone low-temperature heat treatment during pasteurization, making its rapid and accurate detection crucial for ensuring the quality control of dairy products. An enzyme-linked immunosorbent assay-based analytical protocol has often been referred to for the detection of LF in real samples. Signal amplification was accomplished using the streptavidin–biotin system. Here, an automated magnetic beads-based sandwich chemiluminescence enzyme immunoassay (MBs-sCLEIA) system was developed for the quantification of lactoferrin in pasteurized milk. The MBs-sCLEIA system consists of an automated chemiluminescence-based analyzer and a lactoferrin MBs-sCLEIA assay kit. Notably, our proposed method eliminates the need for pretreatment procedures and enables the direct addition of milk samples, allowing for the automatic quantitative detection of lactoferrin within a rapid 17 min timeframe for up to eight samples simultaneously. The MBs-sCLEIA was linear over the range of 7.24–800 ng/mL and displayed a limit of detection (LOD) of 2.85 ng/mL. As its good recovery and CV values indicate, the method exhibited high precision and accuracy. Furthermore, it was verified that it was selective towards five additional common milk proteins. A good correlation was observed between the results from the MBs-sCLEIA and heparin affinity column-HPLC (r^2^ = 0.99042), which proves to be a useful and practicable way of conducting an accurate analysis of lactoferrin in dairy products.

## 1. Introduction

Lactoferrin (LF), a member of the transferrin family, is an iron-binding glycoprotein that plays a crucial role in iron transport. It possesses a relative molecular weight of 80 kDa [1]. LF is predominantly present in mammalian milk and secretions (e.g., tears, small intestinal secretions, joint effusions, and amniotic fluid) [2]. Bovine lactoferrin exhibits higher concentrations in the colostrum with levels exceeding 1 mg/mL compared to normal bovine milk which has lower concentrations ranging from 0.02 to 0.3 mg/mL [3]. Various previous studies have shown that this biological macromolecule exhibits several qualities such as being an antioxidant [4], anti-inflammatory [5], antibacterial [6], and antiviral [7] substance, in addition to performing a wide range of physiological and nutritional functions, such as immune modulation [8] and tumor growth inhibition [9]. Furthermore, LF plays a crucial role in maintaining iron homeostasis [10], while also exhibiting its potential to stimulate cell proliferation and differentiation [11]. Consequently, the oral administration of LF is believed to be good for both infants and adults, and the observed host-protective effects have stimulated its worldwide commercial production. To date, lactoferrin has been used in infant formula [12], health products [13], and beverages [14]. In response to this growing demand, more than 200 tons of LF are industrially extracted from cow’s milk worldwide each year [15]. The Ministry of Health of the People’s Republic of China has clear guidelines concerning the addition of lactoferrin as a food nutrition fortifier in China. Its content must not be added in excess of 1.0 g/kg when making milk, fermented milk, milk beverages, and other dairy products [16]. Therefore, the selection of appropriate detection methodologies for screening and categorizing lactoferrin content in raw milk, as well as confirming its presence in dairy products, hold immense significance for the advancement of the milk and dairy industry.

The detection of lactoferrin has been possible using a variety of techniques over the years, including high-performance liquid chromatography (HPLC) [17], high-performance liquid chromatography–tandem mass spectrometry (HPLC-MS/MS) [18], polyacrylamide gel electrophoresis [19], capillary electrophoresis [20], spectrophotometry [21], and immunological methods [22,23]. However, the pretreatment process for detecting sample solutions in dairy products using high-performance liquid chromatography is challenging and time-consuming due to the complex matrix of dairy products and stringent requirements. Sodium dodecyl sulfate–polyacrylamide gel electrophoresis (SDS-PAGE electrophoresis) fails to meet the demands for large-scale lactoferrin detection in dairy production sites due to its limited sample capacity. Although spectrophotometry provides a quick and easy method to determine lactoferrin content, its detection accuracy is inadequate for quantifying LF in raw milk and dairy products [24]. The immunological method is characterized by its sensitivity, selectivity, convenience, and high throughput capacity [25]. It encompasses the detection of current changes relayed by electrochemical biosensors [26], color, or chemiluminescence signals generated through enzyme reactions, and optical signals detected via surface plasmon resonance (SPR) [27,28]. However, there are several drawbacks associated with their utilization. For instance, designed electrochemical biosensors often necessitate complex electrode surface construction and frequent calibration, washing, and incubation procedures. Consequently, this leads to a protracted and intricate operational process throughout the entire detection procedure [29]. In addition, the temperature of the SPR analysis test has a great influence on the determination results, and the cost of equipment is also expensive. Although a direct competitive enzyme-linked immunosorbent assay (ELISA) has been applied to the detection of lactoferrin in a variety of dairy products [22], a sandwich ELISA is often used to detect macromolecular proteins, having better sensitivity and specificity than the direct competition method [30,31]. Additionally, the biotin–streptavidin system used to be applied to label proteins or nucleic acids for a sensitive determination. A streptavidin molecule (usually extracted from streptomycin) can theoretically react to four biotin molecules with extremely strong specificity and affinity (the dissociation constant K_D_ = 10^−15^ mol/L), which is much higher than that between antigen and antibody [32].

Considering the limitations described and the advantage of using a sandwich ELISA previously outlined, we have developed a magnetic beads-based sandwich chemiluminescence enzyme immunoassay (MBs-sCLEIA) for lactoferrin detection. This method is based on the strong specific affinity between biotin and streptavidin, coupled with alkaline phosphatase-assisted secondary signal amplification. Previous studies have shown that biotin is a B vitamin with thermal stability and is very abundant in milk and dairy products [33,34]. Therefore, to avoid the negative impact of the passive adsorption of lactoferrin antibodies, streptavidin was utilized as a scaffold to directionally immobilize biotinylated LF mAb on the magnetic beads to further capture lactoferrin in milk samples with complex matrices. The alkaline phosphatase catalytic substrate provides the possibility of signal amplification. The chemiluminescence signal is different from the traditional colorimetric signal, which requires a certain reaction time and termination step. Both the quick response time and the noticeable signal change reduced the detection time and increased the sensitivity of the lactoferrin detection. Furthermore, a number of the characteristics of the standard solution or milk samples were assessed, including linearity, accuracy, precision, and selectivity. Lastly, the MBs-sCLEIA was successfully applied to evaluate LF content in dairy products. Consequently, it holds the potential for quality control (QC) in raw milk acceptance, quality supervision, and risk assessment purposes.

## 2. Materials and Methods

### 2.1. Reagents and Apparatus

Standard lactoferrin was bought from Sigma-Aldrich Chemical Co. (St. Louis, MO). Bovine IgG, α-Lactalbumin, β-Lactoglobulin, and casein were bought from Shanghai Yuanye Biotechnology Co., Ltd. (Shanghai, China). Lactoferrin mAb_1_ (3.20 mg/mL) and mAb_2_ (2.70 mg/mL) were purchased from Zhunce. Bio. (Huzhou, China). Streptavidin magnetic beads (SA-MBs) (10 mg/mL) were purchased from BioMag Scientific Inc. (Wuxi, China). Alkaline phosphatase conjugate stabilizer-01 buffer (ACS-01) was bought from Beijing Avid Biotechnology Co., Ltd. (Beijing, China). Ab Stabilizer was purchased from Shandong Lvdu Bio-Sciences & Technology Co., Ltd. (Binzhou, China). Alkaline phosphatase (2 mg/mL), 100 mM Traut reagent, and a PD-10 desalination chromatography column were obtained from Sophonix Co., Ltd. (Beijing, China). Three brands of pasteurized milk were purchased from a local supermarket, including Yili, Mengniu, and Brightdairy. Furthermore, a heparin affinity column was bought from Meizheng Bio-Tech Co., Ltd. (Rizhao, China). Trifluoroacetic acid (TFA) was purchased from Alfa Aesar Chemicals, Co., Ltd. (Shanghai, China). All other reagents were of at least analytical reagent grade and used without further purification. The water used for all experiments was purified using a PURELAB^®^ Chorus 1 Complete (ELGA Lab Water, High Wycombe, UK).

The following buffers were used: a TN buffer mixture of 100 mM of Tris-HCl (pH 7.4); a 1.5 M of NaCl; a TH-1T buffer containing 1 M of Tris-HCl (pH 7.4); 1% Tween 20; MES buffer containing 0.5 M of 2-Morpholinoethanesulphonic acid (pH 6.7); 1 mM of EDTA; a binding buffer mixture of 84 mM of Na_2_HPO_4_ · 12H_2_O and 16 mM of Na_2_HPO_4_ · 2H_2_O; an elution buffer containing 21 mM Na_2_HPO_4_ · 12H_2_O and 4 mM Na_2_HPO_4_ · 2H_2_O; and 500 mM NaCl.

The two labeled anti-lactoferrin antibodies were quantified by the Thermo Nanodrop 2000c spectrometer (Middlesex County, MA, USA). The chemiluminescence intensity was monitored by an Aceso 80A automated chemiluminescent immunoassay analyzing system (Sophonix Co., Ltd., Beijing, China). The chromatographic analysis was performed on an Agilent 1100 HPLC system (Agilent Technologies, Santa Clara, CA, USA) with a Variable Wavelength Detector (VWD) and an AdvanceBio RP-mAb C4 column (150 mm × 4.6 mm, 3.5 μm; Agilent).

### 2.2. Preparation of mAb_1_-Labeled Biotin

Biotinylated mAb_1_ was prepared by coupling the amino group of anti-LF mAb_1_ with the N-hydroxysuccinimide of NHS-biotin with the NHS ester crosslinking reaction. NHS-PEG4-Biotin is a PEGylated, water-soluble reagent, causing reactions with primary amino groups under slightly alkaline conditions (pH 7.2–8.5). In this reaction, stable amide bonds are formed while N-hydroxysuccinimide is released. LF mAb_1_ is crosslinked with biotin by its primary amino group in the N-terminus or by several primary amines in the side chain of lysine. Therefore, NHS-PEG4-Biotin was dissolved in dimethylformamide, and a 20 mM sodium phosphate buffer adjusted to pH 7 was used to dilute it to 10 mM. The crosslinking reaction was started by the addition of 10 mM of NHS-PEG4-Biotin at ambient temperature. After 30 min, the reaction was stopped using an ultrafiltration concentration tube to centrifuge at 8000 rpm for 15 min. The supernatant was collected and added with an equal volume of 50% glycerol; it was then stored at −20 °C until use.

### 2.3. Preparation of mAb_2_-Labeled Alkaline Phosphatase

Firstly, 370 μL of LF mAb_2_ (2.7 mg/mL) was diluted with TES8.5 diluent and passed through the PD-10 desalination chromatography column. Then, 3 mL of the chromatography solution was collected and centrifuged at 8000 rpm for 15 min at room temperature using an ultrafiltration concentration tube. All supernatants were collected and mixed with 100 mM of Traut reagent at a molar ratio of 1:50 at room temperature for 30 min. The NH_2_ group was converted into a SH group using a ring-opening reaction, while maintaining charge properties similar to the original amino group, thereby completing the amino modification of LF mAb_2_. An appropriate amount of 1 M of glycine was added to react for 5 min to terminate the antibody activation.

At the same time, alkaline phosphatase (ALP) (2 mg/mL) was also reacted with 20 mM of 4-(N-maleimidomethyl) cyclohexane-1-carboxylate (SMCC) dissolved in DMF at room temperature for 30 min, and then, glycine was added to terminate the reaction. SMCC is an amine-to-sulfhydryl crosslinker that contains NHS-ester and a maleimide reactive group. As a hetero-bifunctional linker, SMCC was capable of offering an activated carboxyl group on the alkaline phosphatase, covalently reacting with the sulfhydryl group on the antibody. Therefore, the activated LF mAb_2_ and alkaline phosphatase were mixed and reacted overnight at 4 °C for 12 h to complete the labeling of antibodies by alkaline phosphatase. Subsequently, 100 mM of ethanolamine was added to end the reaction, and the product was purified using an AKTA protein purification instrument. Finally, the ALP-LF mAb_2_ was quantified using a NanoDrop spectrophotometer and added with an equal volume of 50% glycerol; it was then stored at −20 °C until use.

### 2.4. Immobilization of the Biotin- Conjugated mAb1 on the Streptavidin Magnetic Beads

Based on the strong specific affinity between streptavidin and biotin, anti-lactoferrin mAb_1_-coupled magnetic beads were prepared. The specific experimental steps were as follows: After sufficient vortex mixing, 50 µL of brown streptavidin magnetic beads were removed in 2 mL centrifuge tubes. Subsequently, different volumes of biotinylated lactoferrin mAb_1_ diluted 1000 times using MES buffer were also added. The MES buffer was used to make up the volume of the reaction system to 600 µL. The mixture was shaken at 37 °C for 30 min. The biotin- conjugated LF mAb_1_-conjugated magnetic beads were separated with an external magnet and the supernatant was carefully removed; then, the beads were washed 3 times with 1 mL of PBS-BSA buffer (PBS containing 5% BSA) and once with 1 mL of MES buffer to remove biotin-free LF mAb_1_ and block nonspecific binding. Finally, the conjugations were resuspended in 1 mL of MES and then stored at 4 °C for further use.

### 2.5. Establishment of MBs-sCLEIA for LF Detection

The procedure for quantitative lactoferrin detection using the MBs-sCLEIA was as follows: 50 µL of SA-MBs-biotinylated LF mAb_1_, 10 µL of LF standard solution, and 50 µL of ALP-LF mAb_2_ were added into the reaction wells of the reagent strips in turn. After being fully mixed at 37 °C, the SA-MBs-biotinylated LF mAb_1_-LF- LP-LF mAb_2_ sandwich complex was formed based on the specific binding of antigen and antibody, and unreacted antibodies were removed by magnetic separation. The sandwich complex was washed three times with TBST (0.1% Tween 20), and then reacted with 150 µL of luminescent substrate APS to produce a chemiluminescence signal. The reagent removal and signal value detection in the whole LF quantitative process were completed using a chemiluminescent immunoassay analysis system.

### 2.6. Data Analysis

Standard LF samples diluted in whole milk that were subjected to ultra-high-temperature (UHT) treatment were measured in triplicate, and the mean values were processed. Standard curves were obtained by plotting the concentration of the analyte with the Origin6.0 professional software(Origin 6.0 patch 4). The standard curve was fitted by linear and four-parameter logistics, respectively. The linear regression equation represented the relationship between the logarithm of lactoferrin and the chemiluminescence signal value.

(1) $$Y=\frac{\left(A-D\right)}{1+{\left(\frac{x}{C}\right)}^{B}}+D$$

where *A* is the response at an infinitely small standard concentration, *D* is the response at an infinite standard concentration, *C* is the IC_50_ (the 50% inhibitory concentration), *x* is the concentration of the analyte, *B* is the curvature parameter that determines the steepness of the curve, and Y is the corresponding response.

### 2.7. Real Sample Detection

The lactoferrin in real samples was confirmed with HPLC, according to the referenced study [35]. The purchased pasteurized milk from 3 brands was pretreated by referring to the heparin affinity column instructions. A pasteurized milk sample (12 mL) with binding buffer (18 mL) was pipetted into a 50 mL centrifugation tube and swirled to mix. The milk sample was then centrifuged at 4 °C and 12,000 rpm for 15 min. The intermediate layer was obtained and enriched using the heparin affinity column. According to the manufacturer’s instructions, the enrichment of lactoferrin from different pasteurized milk pretreatment solutions was performed using a heparin affinity chromatography column (3 mL). The column was first equilibrated at room temperature with 5 mL of binding buffer. A pretreatment solution (10 mL) was then run through the column at a flow rate of 1 mL/min. The column was then washed using 10 mL of binding buffer, thus washing nonspecific binding impurities, followed by sequential elution steps using 4 mL of elution buffer and obtaining a washing solution. The eluent was subsequently subjected to centrifugation at 12,000 rpm for 5 min at room temperature, and the supernatant layer was carefully collected prior to HPLC analysis. Each pasteurized milk sample was diluted 800 times with MES and then detected with the sCLEIA. The reliability of our method was determined by analyzing the samples simultaneously with heparin affinity column-HPLC, and a correlation analysis was conducted by comparing the detected results from the HPLC with those from the sCLEIA.

## 3. Results

### 3.1. MBs−sCLEIA for Lactoferrin

**Figure 1:**
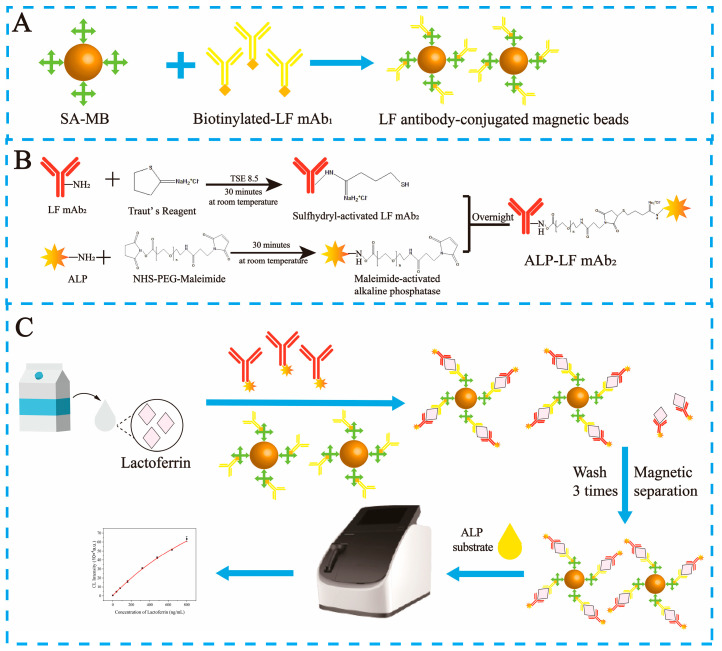
The schematic diagram of MBs−sCLEIA for LF detection. (**A**) The assembly of biotinylated LF mAb_1_- conjugated magnetic beads. (**B**) The LF mAb_2_-labeled alkaline phosphatase. (**C**) The process of detecting LF based on MBs−sCLEIA. (SA-MB: streptavidin magnetic bead; LF: lactoferrin; ALP: alkaline phosphatase).

MBs−sCLEIA lactoferrin detection is a one-step process (Figure 1C): an immune sandwich complex consisting of LF mAb_1_ coupled to streptavidin magnetic beads (Figure 1A), LF mAb_2_ labeled with alkaline phosphatase (Figure 1B), and lactoferrin. The amplification strategy carried out in this study was based on the biotin–streptavidin system. Because there are four biotin-specific binding sites on one streptavidin, more biotin- conjugated LF mAb_1_ was coupled with magnetic beads, thereby increasing the LF mAb_1_ involved in the immune response and fully forming an immune sandwich complex. Furthermore, the accuracy of the MBs-sCLEIA was improved by preparing SA-MBs-biotinylated LF mAb_1_ in advance to avoid the interference of biotin in dairy products. Then, a chemiluminescence signal amplification process was generated through an enzymatic reaction. In the presence of lactoferrin analytes, the sandwich structure of antibody–lactoferrin–antibody could be formed on the magnetic beads (Figure 1A) leading to the catalysis of substrates by the alkaline phosphatase. Once catalyzed, substrates formed an intermediate state in the excited state, resulting in the energesis of the intermediate state to a ground state and thereby producing a significant enhancement of the chemiluminescence signal. The chemiluminescence intensity was positively correlated with the concentration of lactoferrin, so the level of analytes was directly reflected. On the contrary, in the absence of analyses, ALP-LF mAb_2_ was washed away due to the unsuccessful formation of a sandwich structure. Thus, the enzymatic reaction could not be triggered and the change in the chemiluminescence signal was nearly negligible. From this, lactoferrin can be analyzed quantitatively by reading out the chemiluminescence signals.

### 3.2. Optimization of Experimental Conditions

**Figure 2:**
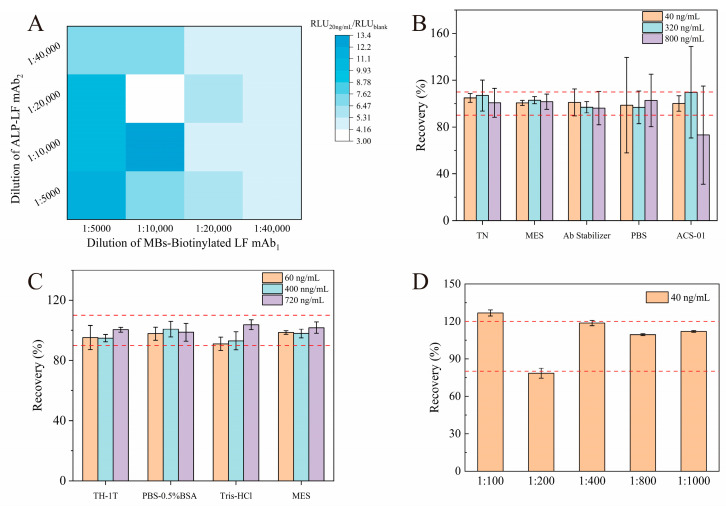
Optimization of experimental conditions. (**A**) The dilution of ALP-LF mAb_2_ and MBs-biotinylated LF mAb_1_. The recovery of lactoferrin in three levels spiked with (**B**) LF mAbs diluting with different buffers (TN, MES, Ab Stabilizer, PBS, and ACS-01) and (**C**) a sample diluting with different buffers (TH-1T, PBS-0.5%BSA, Tris-HCl, and MES). (**D**) The recovery of lactoferrin with different sample dilutions (1:100, 1:200, 1:400, 1:800, and 1:1000). The discontinuous lines correspond to the recovery of the MBs−sCLEIA test within the limits of the acceptable values for the percentages of analyte assayed.

SA-MBs-biotinylated LF mAb_1_ and ALP-LF mAb_2_ were diluted into four concentrations, respectively. The signal value RLU_20 ng/mL_ of the spiked sample and the signal value RLU_blank_ of the negative sample were measured, respectively, and the ratio of RLU_20 ng/mL_/RLU_blank_ was compared. A higher ratio indicated a higher sensitivity. Figure 2A demonstrates that the optimal working dilutions of the SA-MBs-biotinylated LF mAb_1_ and ALP-LF mAb_2_ were both determined to be 1:10,000. Under these conditions, the ratio of 20 ng/mL to the blank chemiluminescence value was the largest, and the sensitivity of the detection method was the highest. Moreover, the suitable diluent can provide a good buffering environment for maintaining the biological activity of SA-MBs-biotinylated LF mAb_1_ and ALP-LF mAb_2_. As shown in Figure 2B, the two antibodies were diluted to the optimal ratio using MES to determine the lactoferrin standard. Compared with the recovery results obtained using four other diluents, the RSD did not exceed 10%, indicating good determination stability. The optimization results of the sample diluent are shown in Figure 2C. The determination results of the recoveries of the low, medium, and high concentrations of the four sample diluents all meet the expected requirements. The samples were diluted using MES, and the RSD of the recovery rates of the different concentrations were not more than 5%, showing good detection accuracy. In addition, in order to better maintain the biological activity of lactoferrin antibody and not introduce other diluents to interfere with its detection sensitivity, the lactoferrin antibody protective solution MES was selected as the diluent of the sample for subsequent experimental operation. The results (Figure 2D) revealed that a sample buffer dilution of 1:800 provided optimal performance, which could meet the lactoferrin detection requirements of the actual sample concentration in the range of 5.79–64 µg/mL. Furthermore, the interference of the complex matrix in milk was also reduced after dilution, which was more conducive to analytical accuracy and repeatability.

### 3.3. Performance of MBs−sCLEIA

**Figure 3:**
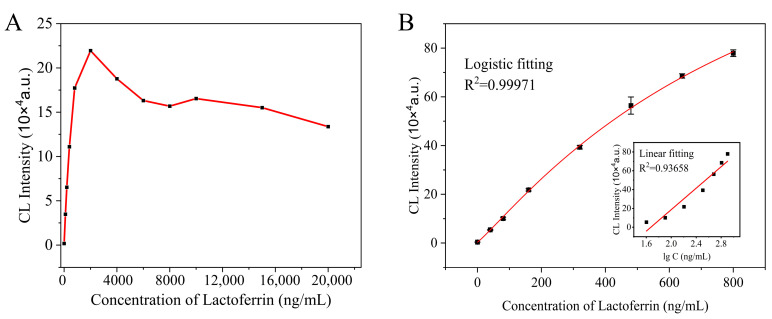
(**A**) The relationship between the lactoferrin concentration and the chemiluminescence signal. (**B**) The relationship between the lactoferrin concentration UHT milk and the CL intensity. Inset is the calibration plot.

We measured the chemiluminescence signal of different concentrations of lactoferrin added to UHT milk. As shown in Figure 3A, the chemiluminescence signal increased with LF concentrations from 0 to 2000 ng/mL. Obviously, the sandwich CLEIA classic “hook effect” appeared when the signal value corresponding to the concentration exceeded 2000 ng/mL. That is, the concentration of lactoferrin is too high, which causes it to bind to the capture antibody and the labeled antibody, respectively, and means it cannot form an effective “sandwich complex”. Therefore, when the concentration of lactoferrin increases, the chemiluminescence signal value decreases instead. Figure 3B illustrates the standard curve obtained from four-parameter logistic fitting and linear fitting models, encompassing lactoferrin concentrations ranging from 0 to 800 ng/mL. The correlation coefficient for the four-parameter logistic fitting was R^2^ = 0.9997, while for the linear fitting, it was R^2^ = 0.93658. Table 1 presents the determination results of the recovery rates for spiked samples using both fitting methods’ standard curves. The calculated recovery rate based on the four-parameter logistic fitting standard curve fell within a range of 97.9% to 101.8%, whereas the data analysis utilizing the linear fitting standard curve yielded a recovery rate between 96.3% and 104.1%. Although there was no significant difference in the recovery rates obtained by these two data fitting methods, it was noteworthy that the correlation coefficient (R^2^ = 0.9997) of the four-parameter logistic fitting standard curve indicated a superior fit quality. Therefore, four-parameter logistic fitting was selected as the model for the standard curve data analysis. The chemiluminescence intensity is higher than the background and the limit of detection (LOD) was calculated to be 2.85 ng/mL based on 3-fold the standard deviation of 20 blank samples (*n* = 3) and the mean. The limit of quantification that was defined as the mean of 20 blank samples plus 10 times the standard deviation was 7.24 ng/mL. The high sensitivity of this mAb-based CLEIA enables us to detect LF in bovine milk powder at a high dilution factor, which greatly brings down possible matrix interferences. A comparison of our MBs−sCLEIA with some previously reported lactoferrin detection methods is presented in Table 2. Most lactoferrin detection methods can only demonstrate one of the advantages of high sensitivity or short detection time. However, our MBs-sCLEIA not only achieves a similar detection limit as previous studies but also completes the entire process within 17 min without requiring complex sample pretreatment; simply diluting the milk sample suffices. This innovative approach offers significant advantages for lactoferrin analysis and represents a promising avenue for future research in this field.

#### 3.3.1. Accuracy and Precision

The accuracy and precision of the MBs−sCLEIA were further evaluated by determining the recovery rates and CVs of three LF-spiked milk samples by conducting intra- and inter-assays. Accuracy refers to the degree of consistency between the measurement result and the measured true value. The standard of LF was diluted in MES to 60 ng/mL, 400 ng/mL, and 720 ng/mL. The intra-assay was performed with three replicates at each concentration on the same day, whereas the inter-assay was completed for three consecutive days. Table 3 shows that the average recovery rates for intra- and inter-assays ranged from 97.9% to 102.2%, and the average CV ranged from 1.2% to 4.3%. Above the recovery rates and CVs demonstrated within the limits of the acceptable values for the percentages of analyte assayed according to AOAC [44], the established method satisfied the need for the quantification of bovine lactoferrin in primary laboratories.

#### 3.3.2. Specificity

**Figure 4:**
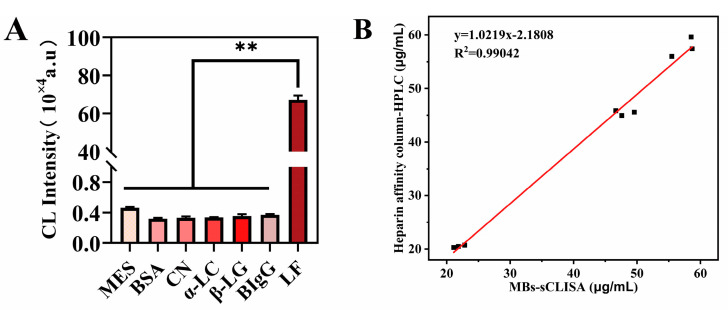
(**A**) Specificity of quantitative LF detection by MBs−sCLISA (*n* = 3). (**B**) Comparison between MBs−sCLISA and heparin affinity column−HPLC in actual pasteurized milk. The red line represents the linear regression analysis of lactoferrin results obtained through two distinct methodologies. (** Mean *p* value < 0.001).

To investigate the specificity of the MBs−sCLEIA, we introduced additional proteins in combination with lactoferrin, i.e., bovine serum albumin (BSA), α-lactalbumin(α-LC), β-lactoglobulin(β-LG), bovine IgG, and casein. Under the optimized conditions, the selectivity of sCLEIA was assessed by the chemiluminescence intensity of lactoferrin with other proteins. The concentration of all milk proteins was set at 200 ng/mL. The results of the assay are shown in Figure 4A; the lactoferrin mAb exhibited high specificity for lactoferrin, and the chemiluminescence intensity of lactoferrin was significantly different from that of other proteins in milk (*p* < 0.001), as determined by a one-way ANOVA analysis of variance. The results of multiple ANOVA comparisons between lactoferrin and other milk proteins are listed in Table 4. It also meant that the slight CL intensity obtained by BSA, casein, α-LC, β-LG, and bovine IgG can result in negligible changes relative to LF mAb. These results indicate that the application of commercial antibodies to the quantitative detection of lactoferrin in real samples is reliable and has great application potential.

### 3.4. Real Sample Analysis

Using nine pasteurized milk samples from three brands, the sandwich CLEIA method was compared with the heparin affinity column−HPLC method. A good correlation between the values of the two methods was found (Figure 4B) and the equation Y = 1.0219X − 2.1808 was derived, where Y was the concentration determined by HPLC analysis and X was that determined by CLEIA; as a result, a correlation coefficient of 0.99042 was calculated. This indicates that the results of the two quantitative methods of lactoferrin have a certain consistency, and a MBs−sCLEIA can be used to quantify lactoferrin in dairy products. Furthermore, the heparin affinity column-HPLC method quantified lactoferrin by the relationship between peak area and lactoferrin concentration, and it took more than 20 min to detect each sample. A MBs−sCLEIA, using the automated chemiluminescent immunoassay analysis system in up to eight samples every 17 min, shortens the detection time and improves the detection efficiency.

## 4. Conclusions

In conclusion, we have successfully developed a sandwich-based immunoassay method that enables the rapid and sensitive detection of lactoferrin in pasteurized milk samples. This method is based on the streptavidin–biotin system and employs an enzyme reaction signal amplification strategy. The recognition element used in this study consists of highly specific and affinity-driven anti-lactoferrin mAb_1_ and mAb_2_ antibodies. By utilizing the streptavidin–biotin system, we were able to immobilize the recognition element onto nano-magnetic beads, allowing for automated operation and significantly reducing the detection time. Additionally, this system synergistically enhances signal amplification through alkaline phosphatase reaction. Experimental conditions such as the dilution ratio of mAb and the sample, as well as the assay buffer, were optimized for the MBs−sCLEIA analysis. Under optimal conditions, the developed MBs−sCLEIA displayed a LOD of 2.86 ng/mL and the linear range was 7.24–800 ng/mL. The developed assay showed a highly sensitive response to lactoferrin. Moreover, the MBs−sCLEIA exhibited many distinct advantages over conventional immunoassays, including a shorter analysis time, a simpler testing process, and a lower number of antibodies. This study indicates the potential of this method to be used to evaluate the quality of milk through the detection of LF based on its high sensitivity and quickness.

## Figures and Tables

**Table 1:** The determination results of the recovery rate for spiked samples using two different fitting methods.

	Spiked (ng/mL)	Recovery (Mean ± SD)
Logistic fitting	60	98.5 ± 1.2%
	400	97.9 ± 2.9%
	720	101.8 ± 3.9%
Liner fitting	60	101.8 ± 0.1%
	400	96.3 ± 0.8%
	720	104.1 ± 1.1%

**Table 2:** Comparison of MBs-sCLEIA with other reported lactoferrin detection methods.

	Materials or Strategy	LOD	Working Range	Test Time	Reference
HPLC	Ion-exchange resin extraction	2 µg /mL	25–514 µg/mL	-	[36]
	HiTrap^TM^ Heparin HP column	2 mg/L	2–100 mg/L	-	[37]
HPLC-MS/MS	Tryptic signature peptides as internal standard	0.3 mg/100 g	10–1000 nmol/L	-	[38]
		0.16 mg/100 g	52.60–150.56 mg/100 g	-	[39]
Capillary electrophoresis	Thermally induced immobilization of poly (2-methyl-2-oxazoline)	5.0 μg/mL	10~500 μg/mL	<10 min	[40]
	CE-Aptasensor	1 nM	4–128 nM	<15 min	[41]
ELISA	Colloidal gold-based strip	9.7 ng/mL	9.76 ~ 625 ng/mL	<15 min	[42]
	AuNFs-based strip	2.4 ng/mL			
	Competitive ELISA	3.9 ng/mL	100–520 ng/mL	>24 h	[22]
Electrochemical detection	Impedance-derived single-frequency capacitive analysis	65.2 nM	125 nM–3.250 μM	<20 min	[27]
	GCE/Nf-MWCNT@MB-LAF involved in the electron-shuttling process to facilitate the H_2_O_2_ reduction reaction	3.2 μM	25 μM–500 μM	30 min	[43]
Surface plasmon resonance transduction	SPR operating in batch mode method	2.8 × 10^−7^ M	0.5–3.50 × 10^−6^ M	>20 min	[28]
	SPR operating in flow mode method	5.0 × 10^−8^ M	0.1–10.0 × 10^−6^ M		
This work	MBs−sCLEIA	2.85 ng/mL	7.24–800 ng/mL	17 min	

- Means not described.

**Table 3:** Accuracy and precision of the MBs-sCLEIA (*n* = 3).

Spiked (ng/mL)	Intra-Assay			Inter-Assay		
	Mean ± SD	Recovery (%)	CV (%)	Mean ± SD	Recovery (%)	CV (%)
60	59.1 ± 0.7	98.5%	1.2%	60.4 ± 1.7	100.6%	2.7%
400	391.8 ± 11.8	97.9%	2.9%	400.7 ± 9.5	100.2%	2.4%
720	732.7 ± 28.1	101.8%	3.9%	735.9 ± 32.0	102.2%	4.3%

**Table 4:** Results of multiple ANOVA comparisons between lactoferrin and other milk proteins.

	MES	BSA	CN	α-LC	β-LG	BIgG
BSA	0.847					
CN	0.860	0.986				
α-LC	0.864	0.982	0.996			
β-LG	0.884	0.962	0.976	0.980		
BIgG	0.901	0.945	0.959	0.963	0.983	
LF	0.000 **	0.000 **	0.000 **	0.000 **	0.000 **	0.000 **

** Mean *p* value < 0.001.

## Data Availability

The original contributions presented in the study are included in the article, further inquiries can be directed to the corresponding authors.
